# Study protocol for ACTIVE study: safety and feasibility evaluation of external ventricular drainage with ACTIVE fluid exchange in intraventricular hemorrhage—a phase 2, multi-center, randomized controlled trial

**DOI:** 10.1186/s13063-022-07043-9

**Published:** 2022-12-29

**Authors:** Mette Haldrup, Niwar Mohamad, Mads Rasmussen, Line Thorup, Stig Dyrskog, Claus Ziegler Simonsen, Rares Miscov, Carsten Reidies Bjarkam, Mads Grønhøj, Frantz Rom Poulsen, Anders Rosendal Korshøj

**Affiliations:** 1 Department of Neurosurgery, Aarhus University Hospital, Palle Juul-Jensens Boulevard 165, 8200, Aarhus N Aarhus, Denmark; 2 Department of Anesthesiology, Section of Neuroanesthesia, Aarhus University Hospital, Aarhus, Denmark; 3 Department of Intensive Care, Aarhus University Hospital, Palle Juul-Jensens Boulevard 165, 8200, Aarhus N Aarhus, Denmark; 4 Department of Neurology, Aarhus University Hospital, Palle Juul-Jensens Boulevard 165, 8200, Aarhus N Aarhus, Denmark; 5 Department of Clinical Medicine, Aarhus University, Palle Juul-Jensens Boulevard 99, 8200, Aarhus N Aarhus, Denmark; 6 Department of Neurosurgery, Aalborg University Hospital, Hobrovej 18-22, 9000, Aalborg, Denmark; 7 Department of Neurosurgery, Odense University Hospital, J.B. Winsløws Vej 4, 5000, Odense, Denmark

**Keywords:** Intraventricular hemorrhage, External ventricular drainage, IRRAflow, Irrigation, Aspiration

## Abstract

**Background:**

Primary intraventricular hemorrhage (IVH) or IVH secondary to intracerebral (ICH) and subarachnoid hemorrhage (SAH) are known to have a very poor prognosis, with an expected mortality between 50 and 80% (Hinson et al. Current Neurology and Neuroscience Reports 10:73–82, 2010). Clearance of IVH might improve patient outcome.

**Methods:**

The study is designed as an investigator-initiated, comparative, prospective, multi-center, 1:1 randomized phase 2 trial evaluating the efficacy and safety of active irrigation in external ventricular drainage (intervention arm—IRRAflow) compared to passive external ventricular drainage (control arm—EVD).

The trial will enroll 58 patients with primary or secondary IVH. Major eligibility criteria include age ≥18 years of age, IVH documented on head CT or MRI scan (Graeb score ≥3), need of cerebrospinal fluid drainage, deterioration of consciousness or medical sedation at the time of enrollment, and indication for active treatment evaluated by the treating physicians. Exclusion criteria included patients with fixed and dilated pupils and pregnant or nursing women.

The primary endpoint of the study is catheter occlusion evaluated by time to first observed occlusion from VC placement. Secondary endpoints include clearance of ventricular blood as measured by head CT scan, rates of catheter-related infection and shunt dependency, length of intensive care unit stay, functional status—Extended Glascow Outcome Scale (eGOS) and modified Rankin scale (mRS) at discharge to rehabilitation and 90 days—and mortality rates at 30 days and 90 days.

**Discussion:**

With no standardized treatment for IVH and a poor prognosis, new treatments are needed. IVH patients often need CSF drainage to treat hydrocephalus and to decrease ICP. Standard treatment with passive external ventricular drainage is related to an increased risk of infections which is found in up to 22% of treated cases. The passive VC is known to have a risk of occlusion and is seen in 19–47% of the cases.

We hypothesize that the use of active fluid change using the IRRAflow system will be safe and feasible and will reduce the occlusion and infection rates in patients with IVH.

**Trial registration:**

ClicalTrials.gov NCT05204849. Registered 15 December 2021. Updated 24 January 2022

**Supplementary Information:**

The online version contains supplementary material available at 10.1186/s13063-022-07043-9.

## Background

Primary intraventricular hemorrhage (IVH) or IVH secondary to intracerebral hemorrhage (ICH) and subarachnoid hemorrhage (SAH) with extension to the ventricles are known to have a very poor prognosis, with expected mortality between 50 and 80% [1]. Yet, therapy directed at ameliorating intraventricular clot has been limited. IVH often results in increased intracranial pressure (ICP) by direct mass effect, by obstruction, and further by creating edema around the hematoma. These conditions can be complicated by hydrocephalus caused by CSF malabsorption [2] or obstruction of CSF pathways. Further secondary brain injury is related to the clotting cascade after endothelial damage and hemoglobin breakdown [3]*.* Thrombin causes inflammatory cells to infiltrate the brain, proliferation of mesenchymal cells, formation of brain edema, and scar tissue [3]*.* Thrombin binds to protease-activated receptors *(1)* and activates the central nervous system microglia and complement cascade. As a result, multiple immune pathways are activated, which contributes to apoptosis and necrosis. Heme influx in neurons after endothelial damage leads to iron release and neuronal insult [3].

CSF drainage is a key factor in maintaining acceptable ICP levels to prevent secondary brain damage. There are multiple approaches to facilitating CSF drainage and monitoring ICP. A ventricular catheter (VC) inserted into the lateral ventricle allows for drainage of CSF, but complication rates are high. VC occlusions in patients with IVH have been reported in 41% [4], of these 19% are having at least one VC replacement [4]. VC-related infection rates are found to be 10% for bacterial ventriculitis [5, 6].

The aim of this study is to evaluate the safety and feasibility in the use of active fluid exchange in the treatment of intraventricular hemorrhage.

## Methods and study design

**Fig. 1:**
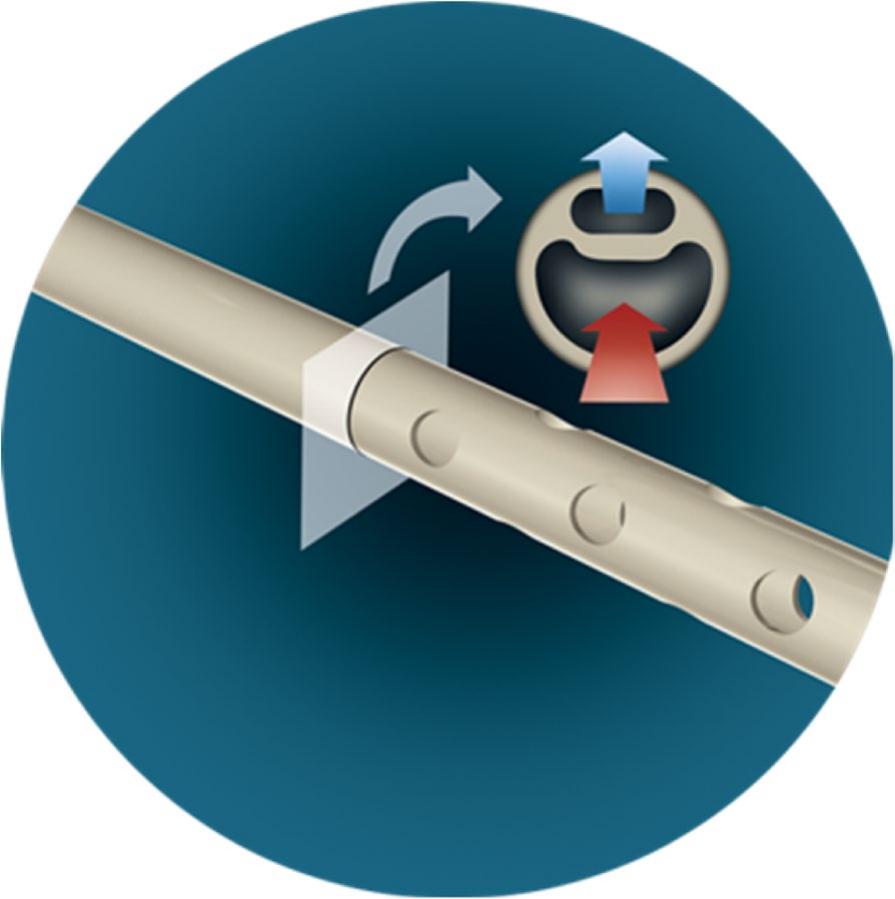
The IRRA*flow* system consists of a dual lumen catheter for irrigation and aspiration respectively (courtesy of IRRAS)

This current phase 2 and feasibility study evaluates the IRRAflow system. A closed system VC with active irrigation and aspiration in comparison to standard passive external ventricular drainage (Fig. 1). We hypothesize that closed system active irrigation will be safe to use and will reduce the occlusion and infections rates and increase clearance rate and clot removal. The framework of the trial is to test the superiority of IRRAflow compared to EVD treatment. This manuscript is based on protocol version 4.0 dated 16th of January 2022. Important amendments to the protocol will be added and shown in clinicaltrials.gov.

**Fig. 2:**
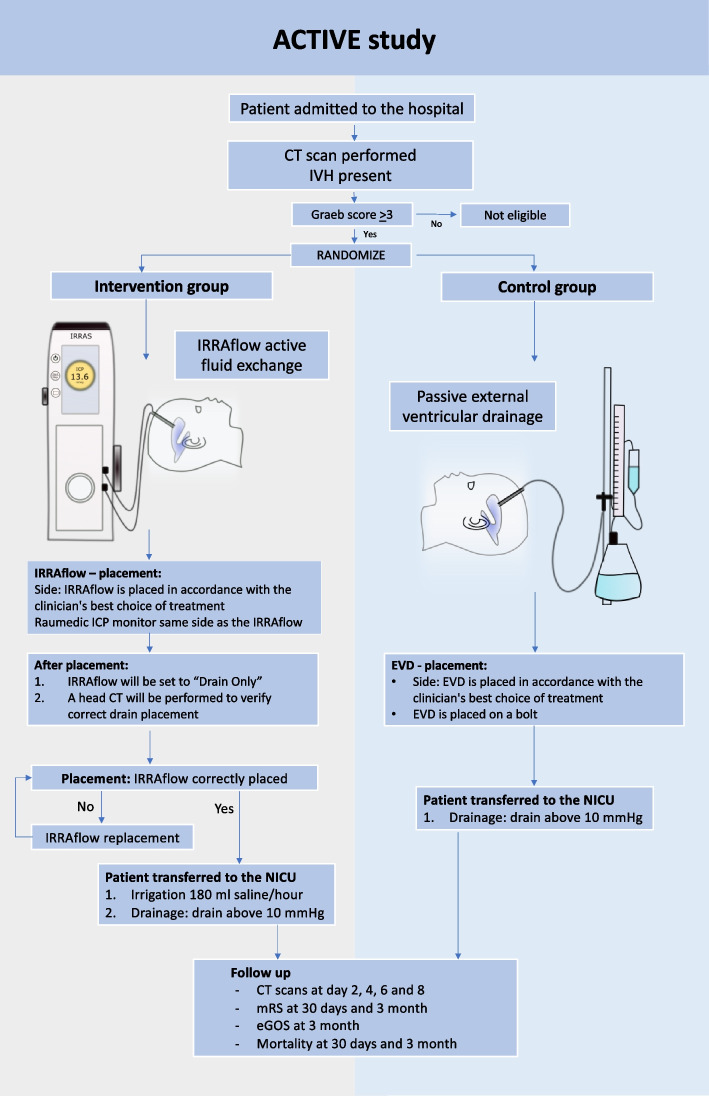
Intervention arm (IRRAflow) and control arm (external ventricular drainage, EVD)

The study is an investigator-initiated, prospective multi-center, 1:1 randomized, comparative phase 2 trial evaluating the efficacy and safety of active irrigation external ventricular drainage (intervention—IRRAflow) compared to passive external ventricular drainage (control—EVD) (Fig. 2). The investigators are unblinded for the treatment, but there is blinding concerning evaluations of endpoints where possible.

The trial will enroll an expected sample size of 58 patients (sample size calculation see “sample size and statistical considerations”). The enrollment period is estimated to be 24 months from the date of the first patient enrolled. Patients will be followed for 3 months with expected trial termination after 27 months from initiation. The study setting is academic hospitals and data will be collected in different neurosurgical departments in Denmark (Aarhus University Hospital and Odense University Hospital).

Intraventricular fibrinolysis (IVF) is prohibited until failure of the catheter. IVF can be administrated after an occlusion of the catheter has appeared.

### The IRRAflow device

The IRRAflow system performs active, controlled fluid exchange, based on the notion that it is faster to wash out IVH, compared to gravity drainage alone. IRRAflow combines periodic, controlled irrigation and aspiration of the catheter probe with neutral physiological fluids. The continuous perfusion cleans the entire inner catheter probe’s surface while the fluid movement helps to disrupt potential clot or bacteria colony formation on the catheter probe’s intracranial external surface, thereby minimizing the problems associated with passive drainage: blockage and infection. IRRAflow perfusion is combined with continuous ICP monitoring that includes safety alarms.

#### Primary endpoint

1. Catheter occlusion evaluated by hours to first observed occlusion from VC placement

#### Secondary endpoints

1. Clearance of ventricular blood measured by the absolute volume of blood in ml on head CT scans
2. Rate of catheter-related infection
3. Length of ICU stay
4. Rate of shunt dependency
5. Functional status—extended Glascow outcome scale (eGOS) and modified Rankin scale (mRS) at inclusion, discharge to rehabilitation and 90 days
6. Mortality rates at 30 days and 90 days

#### Exploratory endpoints

1. Duration of ventricular drainage (EVD and IRRAflow) in days
2. Procedure complications
3. Device-related complications
4. Procedure time
5. Rate of revision procedures of the ventricular catheter
6. Rate of occurrence of repeated hemorrhagic events
7. Number of flushes required
8. Total cost of the procedure

#### Trial overview

The patient population for this study will be comprised of up to 58 patients with the diagnosis of primary intraventricular hemorrhage or secondary IVH from SAH or ICH with an intraventricular breakthrough.

### Ethics

Patient selection criteria are established according to clinical, radiologic, and neurologic components. Patients who meet all the inclusion criteria and none of the exclusion criteria will be eligible for study participation. Candidates for enrolment in the trial will typically be severely disabled by the acute neurological injury occurring due to hemorrhagic stroke. Patients will be in a state of coma or medically sedated and often intubated leaving them physically and/or mentally incapacitated, legally incompetent, and unable to make a proper conscious decision about trial participation and therefore also unable to provide informed consent at the appropriate time of enrollment and guidelines on “acute study” conduct will be followed. The trial investigators will subsequently obtain a written informed consent from the patient or a relative to the patient upon first possible notice in accordance with §§3-5 in the Danish Law on Health Research Ethics (consent form see Additional file 1). No biological samples are taken from the patients nor stored, used, or analyzed in the study and therefore no consent for biological samples is required.

The recruitment strategy is based on a patient enrollment throughout the day. The on-call doctor at each center is instructed in contacting the coordinating investigator when a suitable patient has been admitted to the hospital. The coordinating investigator oversees the randomization of the patient.

#### Inclusion criteria

- Age >18 years of age
- Intraventricular hemorrhage documented on head CT or MRI scan. The scan must be no older than 24 h
- Intraventricular hemorrhage Graeb score ≥3 points [7]
- Need of cerebrospinal fluid drainage (<24 h) deemed by treating physician
- Deterioration of consciousness or under medical sedation at the time of enrollment causing the patient to be mentally and/or physically incapacitated and legally incompetent in the decision of inclusion (“acute study” conduct)
- Indication for active treatment evaluated by the treating physicians
- Use of validated anti-conception for fertile female participants in concordance with guidelines provided by the Danish Health and Medicines Authority or a negative urine HCG test

#### Exclusion criteria

- Patient with fixed and dilated pupils
- Pregnant or nursing women (fertile female participants are required to take a validated pregnancy test for evaluation of pregnancy)

#### The randomization procedure

**Fig. 3:**
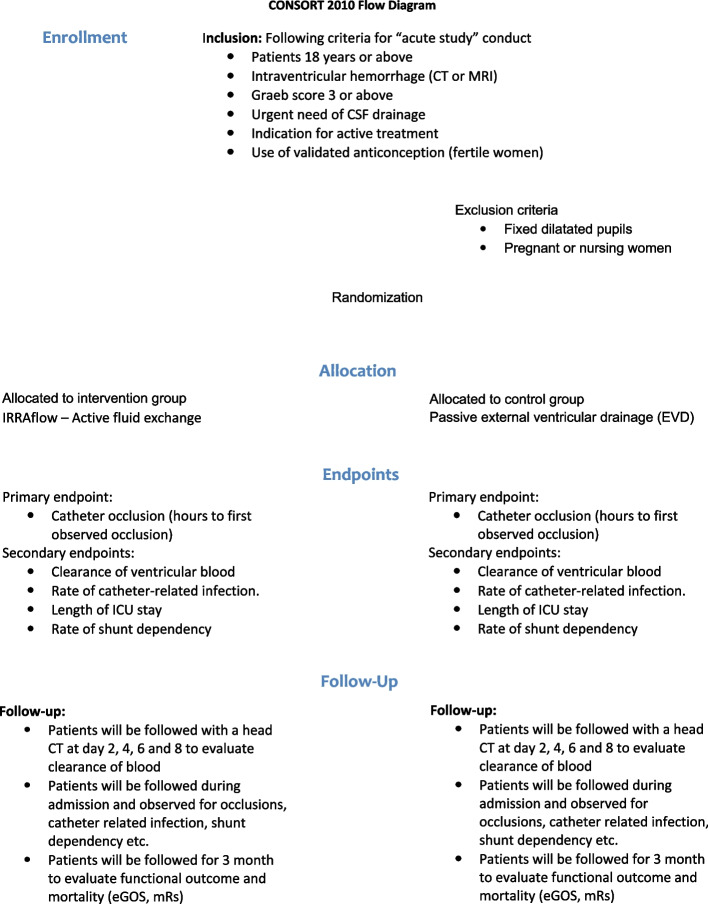
ACTIVE study flowchart

Patients eligible for inclusion following the above-mentioned criteria will be randomized in a 1:1 fashion for EVD or IRRAflow. The allocation sequence is allocated by the RedCAP administrator at Aarhus University. The randomization procedure is performed using the RedCAP randomization module. The randomization is performed by the coordinating investigator in the trial. Randomization will be carried out upon arrival to the operation room and informed consent will be obtained at the first possible occasion after VC placement (Fig. 3).

#### Ventricular catheter placement

**Fig. 4:**
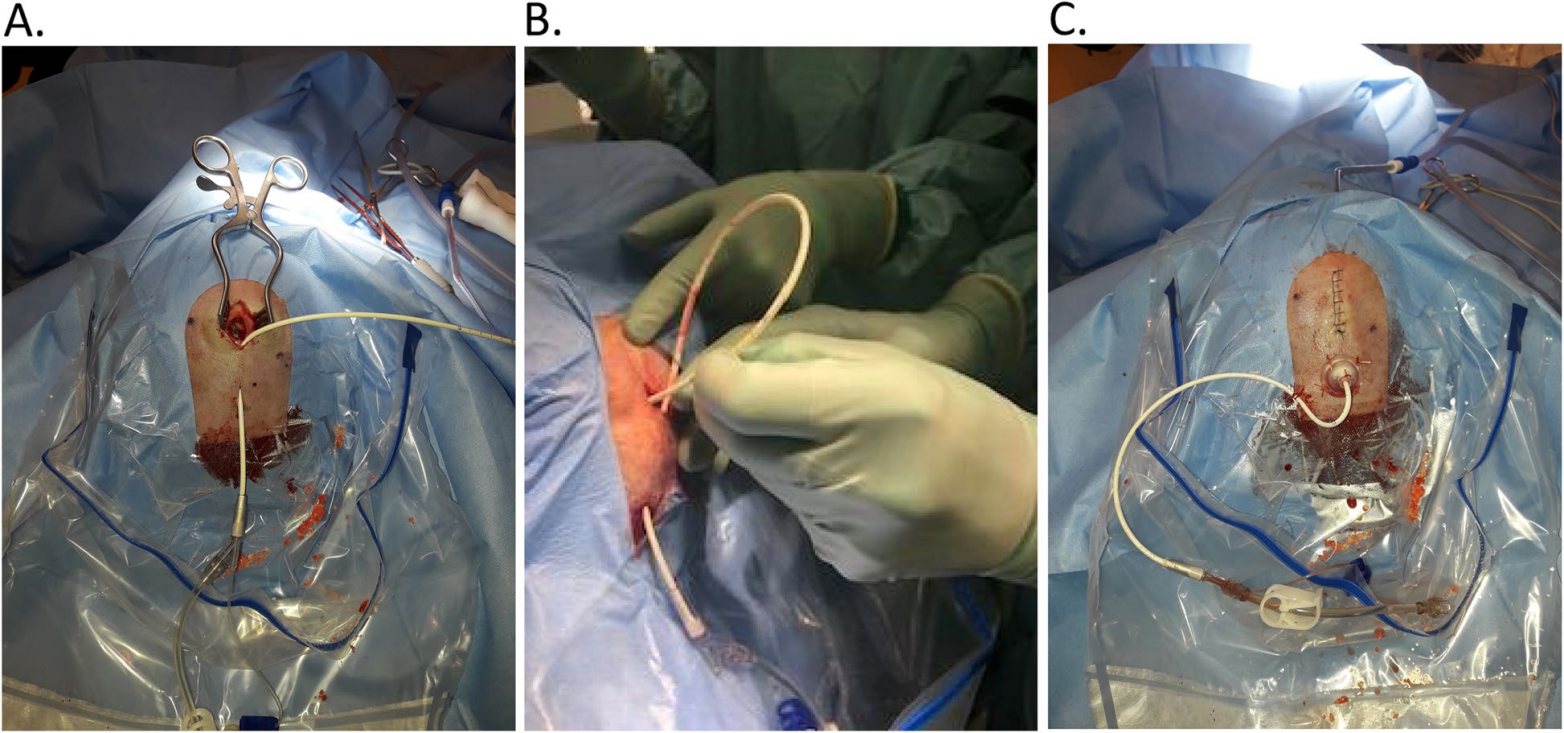
**A** The tunneled ventricular catheter (VC). **B** Placement of the VC in the ventricular system. **C** The result where the VC is attached, and the skin is sutured

VC placement is a standard neurosurgical procedure, and all patients will receive catheters placed according to the standard procedure for EVD placement in both groups. The procedure is performed neuronavigational guided to ensure correct placement and safety for the patient. The catheter is placed in the lateral ventricle containing the least blood and always intended to be placed in CSF outside of the hematoma. The IRRAflow catheter will be placed by reverse tunneling (Fig. 4A–C), whereas the standard EVD will be placed on a bolt.

#### Device training

All investigators, co-investigators, and study personnel will undergo standardized training in study conduct and procedures, including the use and operation of the IRRA*flow* device, prior to study participation. IRRAflow training and certification will be conducted by a qualified IRRAS associate or designee.

#### Follow-up assessments

After placement of the VC, the patients in the two arms will receive the same treatment and monitoring. They will be admitted to the intensive care unit and later transfer to the stationary neurosurgical department before discharge to a rehabilitation facility. Patients will be followed closely regarding the primary, secondary, and exploratory endpoints. During the admission period, the patients will be followed with a CT scan at day 0, day 2, day 4, day 6, and day 8 to evaluate the size of the hematoma using CT volumetrics.

A scheduled follow-up will be applied for all included patients including a clinical status at the time of inclusion, discharge, and 3 months post-procedure. The follow-up will include GCS and mRS at inclusion, discharge, and 3 months. Furthermore, eGOS will be evaluated at discharge and 3 months. The rate of shunt dependency, adverse events, and serious adverse events will be evaluated continuously during the inclusion period. Mortality rates will be evaluated at 30 days and 3 months.

All patients will receive best practice care following trial exclusion or termination. Post-trial care will be independent of trial participation and similar to the care offered to patients who were not enrolled or withdrew consent. Patients who are harmed during the trial will not be offered compensation, which is specific for the trial. However, patients might be eligible for compensation under similar rules and conditions as patients who are not enrolled in the trial as defined by Danish law (Patienterstatningen).

#### Patient withdrawal and lost to follow-up

A patient or patients relative may elect to withdraw from this clinical study at any time. The patient or the patient’s relative should notify the investigator of the request to withdraw. The investigator should encourage patients to return for all required follow-up visits and request that they return for the withdrawal visit.

All patients who withdraw from the study will complete an end-of-study visit. No further visits will be required by the patient once the end-of-study visit is complete. A patient has the right to withdraw from the trial at any time and for any reason without prejudice to his or her future medical care by the physician or the institution. Trial withdrawal by a patient or their LAR specifically means withdrawal of consent from further participation in the trial. Patients who withdraw consent after enrollment will be evaluated to the time of withdrawal, and withdrawal of consent precludes any further trial-related treatment or data collection. LAR may also withdraw consent. At a minimum, every effort should be made to document patient outcome at the time of trial withdrawal.

All patients will be expected to continue in the trial through the final follow-up assessment except in the event of death or upon the patients’ or patients’ relative written request for early withdrawal from the clinical trial.

#### Data management

In the ACTIVE study, all data will be entered electronically and stored in the RedCAP database. Data will be obtained at the site treating the patient and entered directly into the electronic database. Original study forms will be kept in a file at the participating site. Confidentiality of personal information regarding enrolled patients will be kept securely at the study site. All local databases will be secured by password-protected access systems.

#### Sample size and statistical considerations

The sample size of the study was determined based on the primary outcome. We considered a two-sided log-rank test for comparison of the time-to-catheter occlusion between the two treatment groups. Furthermore, we assumed an equal length of follow-up together with an occlusion risk of 10% and 35% in the intervention and control groups. Patient mortality was assumed to be independent of the considered time to catheter occlusion and to correspond to a 30% dropout. We used a significance level of 20% due to the exploratory nature of the study. Then, for a power of 80%, Schoenfeld’s approach resulted in a required sample size of 58 (i.e., 29 subjects with drain per treatment group).

Data will be analyzed according to the intention-to-treat principle. For the primary outcome (time to catheter occlusion), time-to-event analysis will be used, in which drains are considered the basic experimental units. Times corresponding to drains without occlusion during follow-up will be considered censored. Especially, since catheter occlusion is independent of patient mortality, patient death is treated as a censoring event. Time to occlusion will be described by Kaplan-Meier plots and investigated by Cox regression. Estimated beneficial treatment effects with a two-sided *p*-value ≤ 20% will be considered an interesting finding worth further investigation.

Further outcome measures (secondary/exploratory) will be used for descriptive and exploratory purposes only; no formal statistical hypotheses will be tested.

Secondary endpoints (subject-based unless otherwise specified) will be handled as follows:

Kaplan-Meier estimates and Cox regression will be used to describe the time to recurrence of hemorrhage and time to death (with a follow-up time of 3 months).

Occurrence of catheter-related infections and shunt dependency will be reported as proportions, and length of stay in ICU will be reported as means. Clearance of ventricular blood will be described with the help of linear mixed effect models. Measures of functional status will be dichotomized and analyzed by baseline-adjusted logistic regression. All estimates will be reported together with corresponding confidence intervals (CIs).

Exploratory endpoints (mainly drain-based) will be handled as follows:

Proportions and CIs will be reported for technical success, procedure success, and necessity of revision procedures. Procedure time, duration of VC in place, and number of required flushes will be described by means and CIs.

Reported CIs will include a Bonferroni adjustment to maintain the family-wise coverage probability at 95%.

We expect based on our prior experiences that the proportion of missing values in this study will be very low and that missing values occur completely random. All statistical analyses will be run as complete case analyses.

For early safety monitoring, an interim analysis is done when 20 subjects have been enrolled in the study (10 subjects per arm). The analyses will be performed in an intent-to-treat fashion.

Monitored safety outcomes are mortality as well as the total number of adverse and serious adverse events related to catheter treatment (infections, bleedings in relation to intervention, displacements of catheters, catheter misplacement). For *p*-values below 20%, an early stop of the study will be considered (non-binding); otherwise, the study continues until the study end.

#### Data monitoring committee

An Independent Data Monitoring Committee (DMC) has been created with the main purpose of patient safety. The DMC will achieve this by monitoring especially adverse events and severe adverse events and further analyzing the benefit vs risk ratio of the treatment. The DMC will evaluate the safety of the study after the enrollment of 10 patients. The safety evaluation will be based on severe adverse events in each group. For *p*-values below 20%, early stop of the study will be considered (non-binding). Additionally, the DMC will provide an independent scientific review of the interim analysis and recommend continuation or discontinuation of the trial. If the trial passes interim analysis, the DMC will review the final data as well. The DMC will serve in an advisory capacity to the sponsor.

#### Trial steering committee

The Trial Steering Committee (TSC) will consist of the sponsor-investigator and two representatives from each study site. The TSC will act in an advisory capacity to the sponsor in terms of reviewing the progress of the trial and, if necessary, recommend amendments to the protocol or trial logistics to ensure optimal trial progress. Furthermore, the recommendations provided by the DMC will be discussed and, if needed, implemented by the TSC.

#### Publication

All results will be published in peer-reviewed, preferably open-access, international scientific journals and presented at international scientific conferences, regardless of academic conclusions. Positive, negative, and inconclusive results will be publicly available.

## Discussion

With no standardized treatment for IVH and a poor prognosis, new treatment modalities or methods are highly needed. We present a randomized, interventional, clinical phase 2 trial testing a new and innovative intervention, active fluid exchange using the IRRAflow closed irrigation system in comparison to the standard passive external VC for IVH Graeb score 3 or above.

The importance of CSF drainage in patients with IVH is well known in the treatment of acute hydrocephalus and to decrease ICP. Removal of potential cytotoxic material is an added benefit. However, standard treatment with passive external ventricular drainage is known to be related to an increased risk of infections such as ventriculitis and is found in up to 22% of the cases treated with EVD [8, 9]. Furthermore, the passive VC is known to have a risk of occlusion in IVH patients due to blood clotting and is seen in 19–47% of the cases [8].

Previous studies have investigated the use of fibrinolytics to help the removal of the blood in the ventricles. Data from the CLEAR II trial showed that low-dose rt-PA for the treatment of ICH with IVH had an acceptable safety profile compared with placebo and prior historical controls [9]. In the CLEAR III trial, removal of IVH was investigated using either alteplase or saline irrigation [2]. In this study, irrigation of the ventricles with alteplase via a routine EVD did not improve functional outcomes in patients with IVH. One-hundred-eighty-day case fatality was significantly lower in the alteplase group; however, most of these survivors ended up with severe disability (mRS 4 or 5 or eGOS lower and upper significant disability) [10]. The authors also found an association between the amount of clot removal and improved odds of mRS ≤3. The authors concluded that precise clinical definitions for the at-risk population need to be tested in a surgically standardized trial setting and further that a greater benefit could potentially be achieved with greater clot removal (e.g., increased number of patients with >80% removal). They also concluded that future investigation needed to improve the surgical placement of catheters to achieve effective clot reduction more frequently and more rapidly [10]. The IRRAflow system presents a novel approach to minimally invasive clot removal, which is inherently different from alteplase administration through standard EVDs. The continuous irrigation system allows for significant acceleration of IVH clearance as well as other toxins and cellular debris that may accumulate secondary to hemorrhagic stroke. For this phase 2 clinical trial, our main goals will be to evaluate the safety, efficacy, and feasibility of using the IRRAflow active fluid exchange system.

Different new innovations in IVH including novel procedural techniques, the use of the Integra Surgiscope, the use of the Artemis evacuator, the use of BrainPath, novel catheter technology, large bore external ventricular drains, and the CerebroFlo are techniques being tested to lower the morbidity and mortality in IVH patients [11].

Possible risks following the use of active irrigation could be hydrocephalus and increased ICP due to the constant supply of saline. To reduce the risk, saline infusion will be decreased when ICP is found to be 20 mmHg or above or stopped if ICP is still increasing regardless of the decrease in irrigation.

The limitations of the study will be the small differences in the surgical procedures between the two interventions (IRRAflow vs standard EVD). The IRRAflow system is placed using reverse tunneling and is fastened using sutures. The standard passive EVD is placed and fixated through a bolt in the cranium which in former studies is found to reduce the risk of infections and displacements of the VC [12–14].

Furthermore, due to the nature of the surgery and how the IRRAflow device functions, blinding of the surgeons or patients to the intervention is not possible nor a trial design including a sham device or surgery.

## Trial status

Patient enrollment was set of at the 13th of January 2022. On the 20th of May 2022, 10 patients have been included, 5 in each arm. The first safety evaluation meeting in DMC will be held in June 2022 to evaluate AEs and SAEs in the groups.

## Supplementary Information

**Additional file 1. **Substitute consent for participation in a health science research project.

**Additional file 2. **Reporting checklist for protocol of a clinical trial.

## Data Availability

The datasets used and/or analyzed during the current study are available from the corresponding author on reasonable request.
