# Supplementary material for: Study protocol for ACTIVE study: safety and feasibility evaluation of external ventricular drainage with ACTIVE fluid exchange in intraventricular hemorrhage—a phase 2, multi-center, randomized controlled trial
Source: Trials. 2022 Dec 29;23:1062. doi: 10.1186/s13063-022-07043-9 (PMC9798588; doi:10.1186/s13063-022-07043-9)
Supplement: Supplementary file 1 — Additional file 1. Substitute consent for participation in a health science research project. [file 13063_2022_7043_MOESM1_ESM.docx]

**Substitute consent for participation in a health science research project.**

I have received written and oral information and I know enough about the purpose, method, advantages and disadvantages to give my consent.

I know that participation is voluntary and that I can always withdraw my consent without the subject losing his current or future rights to treatment.

I give my consent for ______________________________________ (subject's name) to participate in the research project and I have received a copy of this consent form and a copy of the written information about the project for own use.

Information about my connection, as a relative, to the subject:

________________________________________________________________________________

The name of the person giving proxy consent:_______________________________

Date Signature: ______________________________________________

Would you like information about the results of the research project?:

Yes _____ (set x) No _____ (set x)

Declaration by the person submitting the information:

I declare that oral and written information about the trial has been provided.

The name of the person who submitted the information:

Date Signature: ______________________________________________
